# Assessment of Prognostic Factors and Adjuvant Treatment Modalities in Adult Head and Neck Soft Tissue Sarcoma Patients Treated With Upfront Surgery

**DOI:** 10.7759/cureus.13324

**Published:** 2021-02-13

**Authors:** Baran Akagündüz, Tugba Akin Telli, Sema Sezgin Goksu, Hasan Cagri Yildirim, Muhammet Ozer, Sabin Göktaş Aydin, Neslihan Ozyurt, Cengiz Karacin, Semra Paydas, Mutlu Dogan

**Affiliations:** 1 Medical Oncology, Erzincan Binali Yıldırım Üniversitesi Mengücek Gazi Hastanesi, Erzincan, TUR; 2 Medical Oncology, Marmara University Medical School, Istanbul, TUR; 3 Medical Oncology, Akdeniz University Medical School, Antalya, TUR; 4 Medical Oncology, Hacettepe University Medical School, Ankara, TUR; 5 Internal Medicine, Capital Health Regional Medical Center, Trenton, USA; 6 Medical Oncology, Medipol Unıversity Medical School, Istanbul, TUR; 7 Medical Oncology, Giresun Research Hospital, Giresun, TUR; 8 Oncology, Recep Tayyip Erdoğan University Medical School, Rize, TUR; 9 Medical Oncology, Cukurova University Faculty of Medicine, Adana, TUR; 10 Medical Oncology, Ankara Dr Abdurrahman Yurtaslan Oncology Training and Research Hospital, Ankara, TUR

**Keywords:** survival analysis, head and neck sarcoma, adjuvant radiation therapy, head and neck cancer surgery, adjuvant chemotherapy

## Abstract

Objectives

Head and neck soft tissue sarcomas (HNSTSs) are a heterogeneous group of rare tumors. Surgical resection with negative margins remains the standard primary treatment for patients with HNSTS. The role of chemotherapy (CT) and radiotherapy (RT) remains controversial. In this multicenter study, we aimed to demonstrate the real-world assessing prognostic factors and the effect of adjuvant treatment modalities in adult patients with HNSTS treated with upfront surgery.

Methods

We included a total of 47 patients who underwent curative-intent resection of a primary HNSTS between 2000 and 2019.

Results

The median follow-up was 29 months. The median age of patients was 51 years, and 66% of patients were male. The median relapse-free survival (RFS) of the study population was 31 months (range: 1.0-61.1 months), and the median overall survival (OS) was 115 months (range: 60.8-169.2 months). The univariable analysis revealed that treatment modalities showed a significant impact on RFS (p = 0.021); however, no difference was found in its impact on OS (p = 0.137). R0 resection did not showed impact on RFS (p = 0.130), but a significant association was found with OS (p = 0.004). In multivariable analysis, T stage of the tumor (hazard ratio [HR]: 3.834; 95% CI: 1.631-9.008; p = 0.002) and treatment with surgery and sequential RT and CT (HR: 0.115; 95% CI: 0.035-0.371; p < 0.001) were independent factors associated with RFS. R0 resection was independently associated with OS (HR: 4.902; 95% CI: 1.301-18.465; p = 0.019).

Conclusion

Our study revealed that R0 resection improved OS, and T3-4 stage of tumor was a negative independent factor for RFS in surgically resected HNSTS patients. The use of sequential CT and RT after resection was associated with a better RFS, which emphasizes the importance of multidisciplinary evaluation of the treatment of HNSTS. Randomized prospective studies are needed

## Introduction

Sarcomas are a rare and heterogeneous group of malignant neoplasms derived from mesenchymal tissues. They constitute approximately 1% of all malignancies in the head and neck region [1]. Surgery is the primary treatment of head and neck soft tissue sarcoma (HNSTS). Surgical removal of HNSTS cannot achieve the ideal “wide” resection margins due to their proximity to vital structures and the relatively small space of the head and neck region [2]. Sarcomas generally do not occur in the head and neck region; however, in patients with HNSTS, local recurrence mainly causes mortality [3]. For certain sarcomas, including rhabdomyosarcoma, Ewing sarcoma, and osteosarcoma, chemotherapy (CT) has emerged as a highly effective treatment modality [4-6]. The efficacy of the CT in the neoadjuvant or adjuvant setting for other histologies remains controversial [7-9]. Radiotherapy (RT) is an essential component of multimodality therapy, especially in treating patients with high grade or positive margins following surgical resection [2,10,11]. HNSTSs are rare diseases, and the current literature mainly consists of retrospective and small case series. This study aimed to determine the prognostic factors and the efficacy of postoperative adjuvant treatments in 47 HNSTS patients treated with upfront surgery.

## Materials and methods

Study population

This retrospective study included 47 patients diagnosed with HNSTS between 2010 and 2020 at 10 experienced medical oncology departments in Turkey. Patients who underwent curative-intent resection of a primary HNSTS without neoadjuvant therapy were included in the study. Exclusion criteria were aged < 18 years, metastatic disease at diagnosis, treatment with neoadjuvant CT or RT for locally advanced disease, and patients with the diagnosis of Ewing’s family sarcoma, alveolar or embryonal rhabdomyosarcoma, desmoid-type fibromatosis, and osteosarcoma. The patients receiving adjunctive RT with less than the radical dose (45 Gy) were excluded. The wide excision and radical neck dissection were performed in all patients. The patients with lymph node involvement were excluded. The patients with missing data and those who had secondary primary cancer were also excluded.

Data collection

Data were retrieved from prospectively maintained databases in place at each participating institution. Clinical and demographic features including age, gender, histological subtype, pathological grade according to the FNCLCC (Fédération Nationale des Centres de Lutte Contre Le Cancer) grading system, surgical margin status, tumor size, stage according to the American Joint Committee on Cancer [AJCC] 8th Edition, necrosis, lymphovascular invasion, and the presence of adjuvant RT, CT, or both. Tumor margins were classified as complete (R0) or incomplete (R1/R2). Overall survival (OS) was defined as the time from the diagnosis to death or last follow-up. The relapse-free survival (RFS) was defined as the time from the diagnosis to the relapse or death.

Ethical consideration

This multicenter retrospective study was performed per the Declaration of Helsinki. It was reviewed and approved by the Ethics Committee of the University of Erzincan Binali Yıldırım University School of Medicine.

Statistical analysis

IBM SPSS 25 (Statistics Program for Social Scientists, IBM Corp., Armonk, NY, USA) was used for statistical analysis. Kolmogorov-Smirnov test was used for the compatibility of the data to normal distribution. Non-parametric continuous data were presented as median (range), and categorical data were presented as frequency (percentage). Survival analysis was performed using the Kaplan-Meier method. Log-rank test was used to compare survival times between groups. The independent prognostic factors for OS and RFS were determined using the Cox regression analysis. The time from diagnosis to death due to any reason was defined as OS, and the time from diagnosis to disease relapse or death was defined as RFS. All statistical tests were performed bilaterally, and p < 0.05 was considered statistically significant.

## Results

Clinicopathological features

A total of 47 patients diagnosed with HNSTS between 2010 and 2019 were included in this study. The median follow-up was 29 months (range: 8-163 months). Patients’ characteristics are described in Table 1. Of the patients, 16 (34%) were female and 31 (66%) were male. The median age of patients was 51 years (range: 18-85 years). Of the patients, 42.5% had grade 3 disease, 29.8% had grade 2 disease, and 27.7% had grade 1 disease. Also, 14.9% of patients had stage T1 disease, 27.7% had stage T2 disease, 51.1% had stage T3 diseases, and 6.3% had stage T4 disease. Necrosis was found in 17% of the patients, and vascular invasion was found in 55.3% of patients. In our cohort, 25.2% of patients had synovial sarcoma, 12.6% had leiomyosarcoma, 12.6% had myxofibrosarcoma, 12.6% spindle cell sarcoma, 8.4% angiosarcoma, 6.3% had undifferentiated pleomorphic sarcoma, and 21% of patients had other histological types. Also, 50.3% of patients had neck sarcoma, 19.1% had scalp/face sarcoma, 17% had supraclavicular sarcoma, and 10.7% had paranasal sinus sarcoma.

**Table 1:** Patients’ and treatment characteristics CT, chemotherapy; IMA, ifosfamide and adriamycin; LVI, lymphovascular ınvasion; OS, overall survival; RFS, relapse-free survival; RT, radiotherapy

	n = 47
Age, year, median (range)	51 (18-85)
Gender, n (%)	
Male	31 (66.0)
Female	16 (34.0)
Tumor grade, n (%)	
1	13 (27.7)
2	14 (29.8)
3	20 (42.5)
Necrosis	
Yes	8 (17.0)
No	39 (83.0)
LVI	
Yes	26 (55.3)
No	21 (44.7)
T stage, n (%)	
T1	7 (14.9)
T2	13 (27.7)
T3	24 (51.1)
T4	3 (6.3)
R0 resection, n (%)	
Yes	36 (76.6)
No	11 (23.4)
Treatment modality, n (%)	
Surgery	14 (29.8)
Surgery + RT	6 (12.8)
Surgery + CT	10 (21.3)
Surgery + RT + CT	17 (36.1)
Histological subtypes, n (%)	
Synovial sarcoma	12 (25.2)
Leiomyosarcoma	6 (12.6)
Myxofibrosarcoma	6 (12.6)
Spindle cell sarcoma	6 (12.6)
Angiosarcoma	4 (8.4)
Undifferentiated pleomorphic sarcoma	3 (6.3)
Others	10 (21.0)
Tumor location	
Neck	25 (53.2)
Scalp/face	9 (19.1)
Supraclavicular	8 (17.0)
Paranasal sinus	5 (10.7)
Chemotherapy regimen	
IMA	24 (51.1)
Paclitaxel	1 (2.1)
Ifosfamide	1 (2.1)
Adriamycin	1 (2.1)
None	20 (42.6)

Treatment

In regard to treatment, 29.8% of patients were treated with surgery alone, 12.8% with surgery and adjuvant RT, 21.3% with surgery and adjuvant CT, and 36.1% with surgery and sequential CT and RT. Also, 51.1% of patients were treated with ifosfamide and adriamycin, 2.1% with ifosfamide, 2.1% with adriamycin, and 2.1% with paclitaxel.

Survival analysis

**Figure 1:**
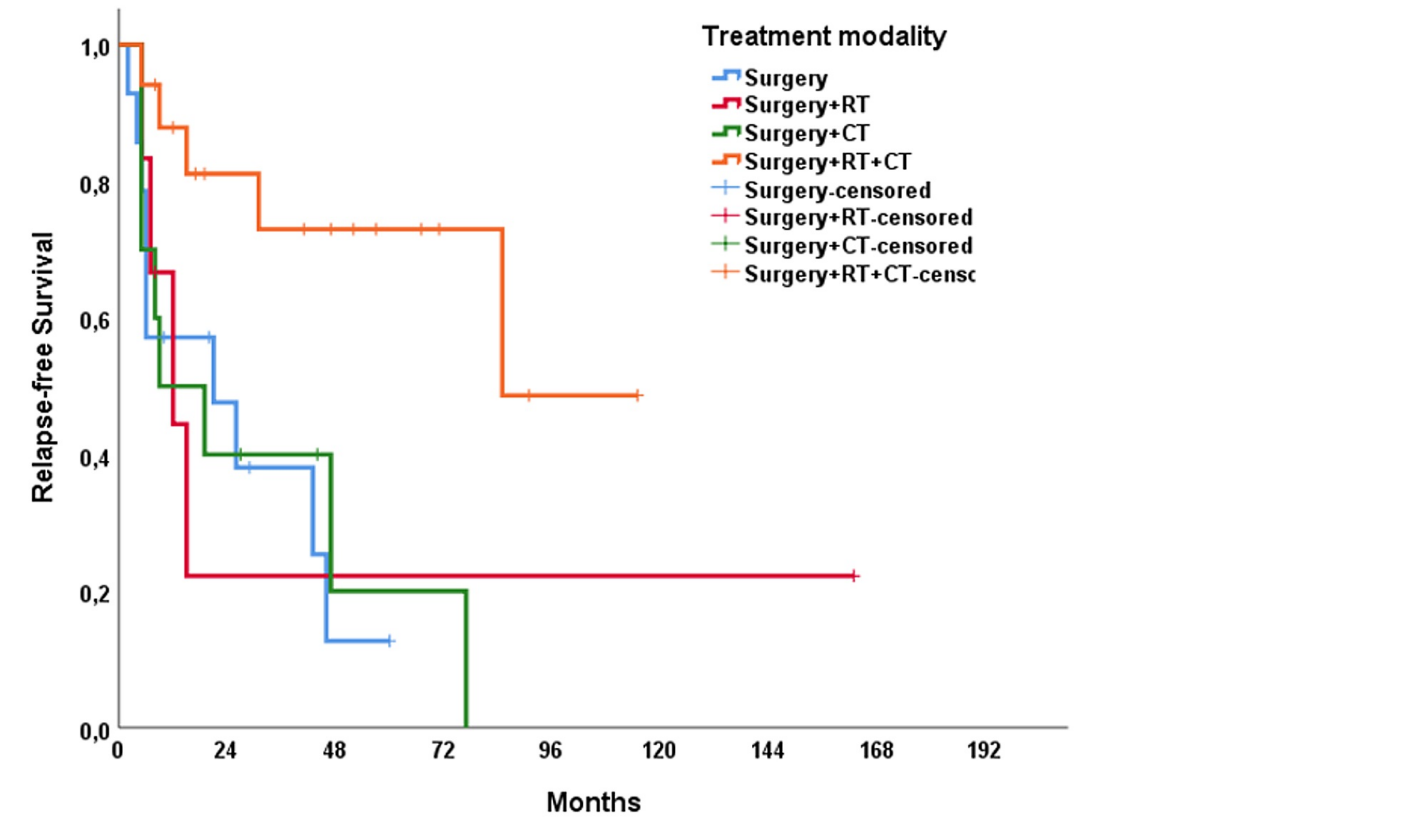
Relapse-free survival by treatment modalities

**Figure 2:**
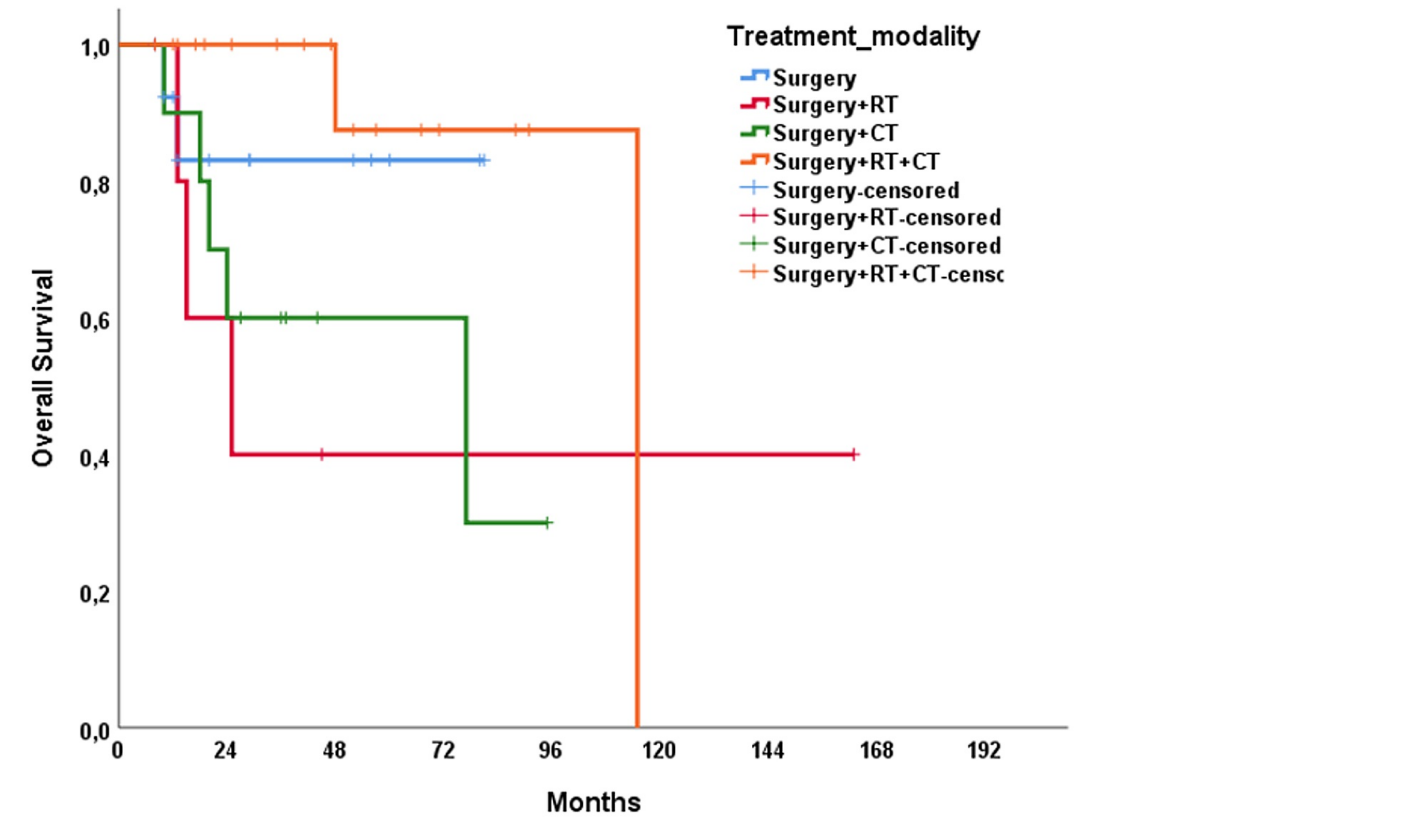
Overall survival by treatment modalities

**Figure 3:**
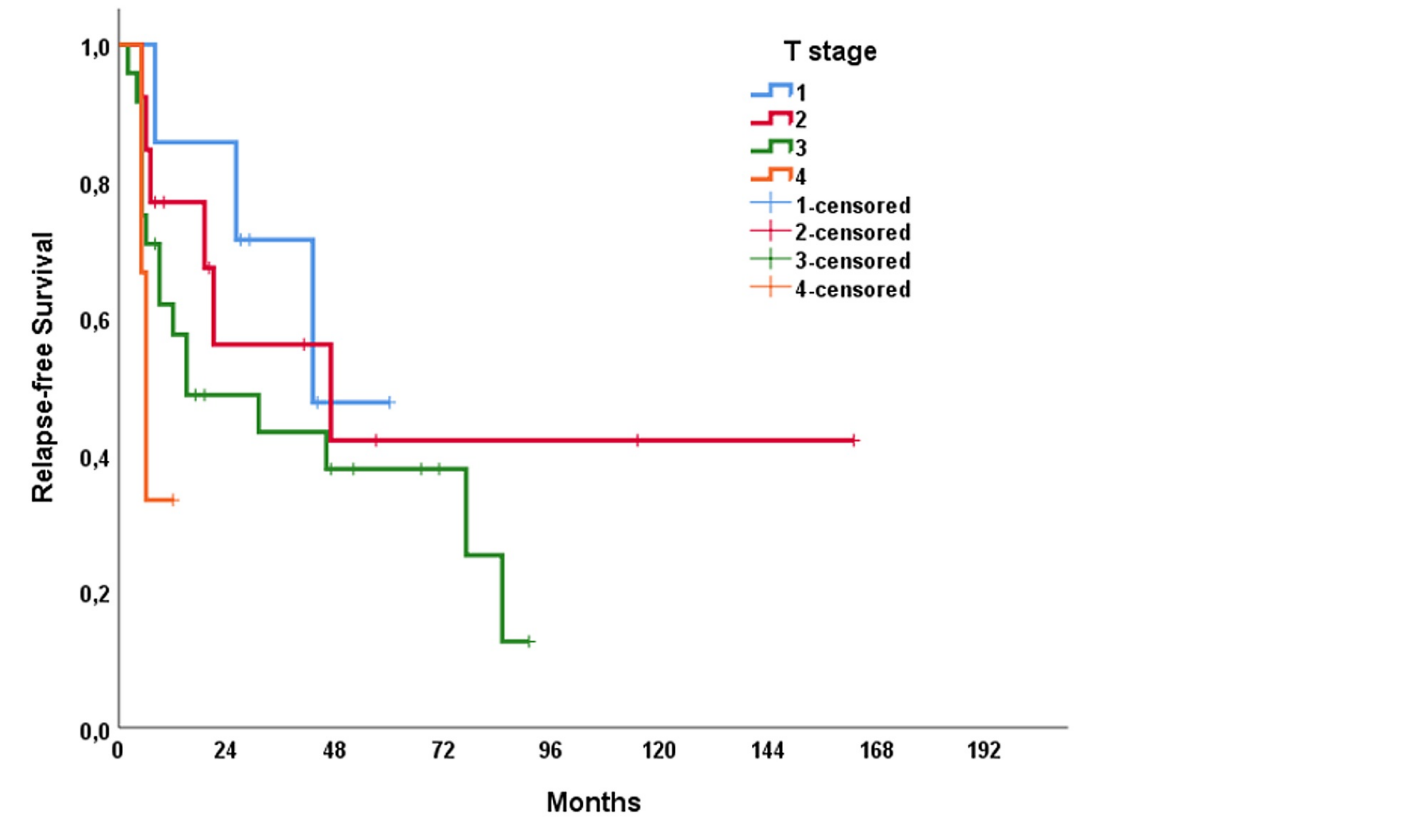
Relapse-free survival by T stage

**Figure 4:**
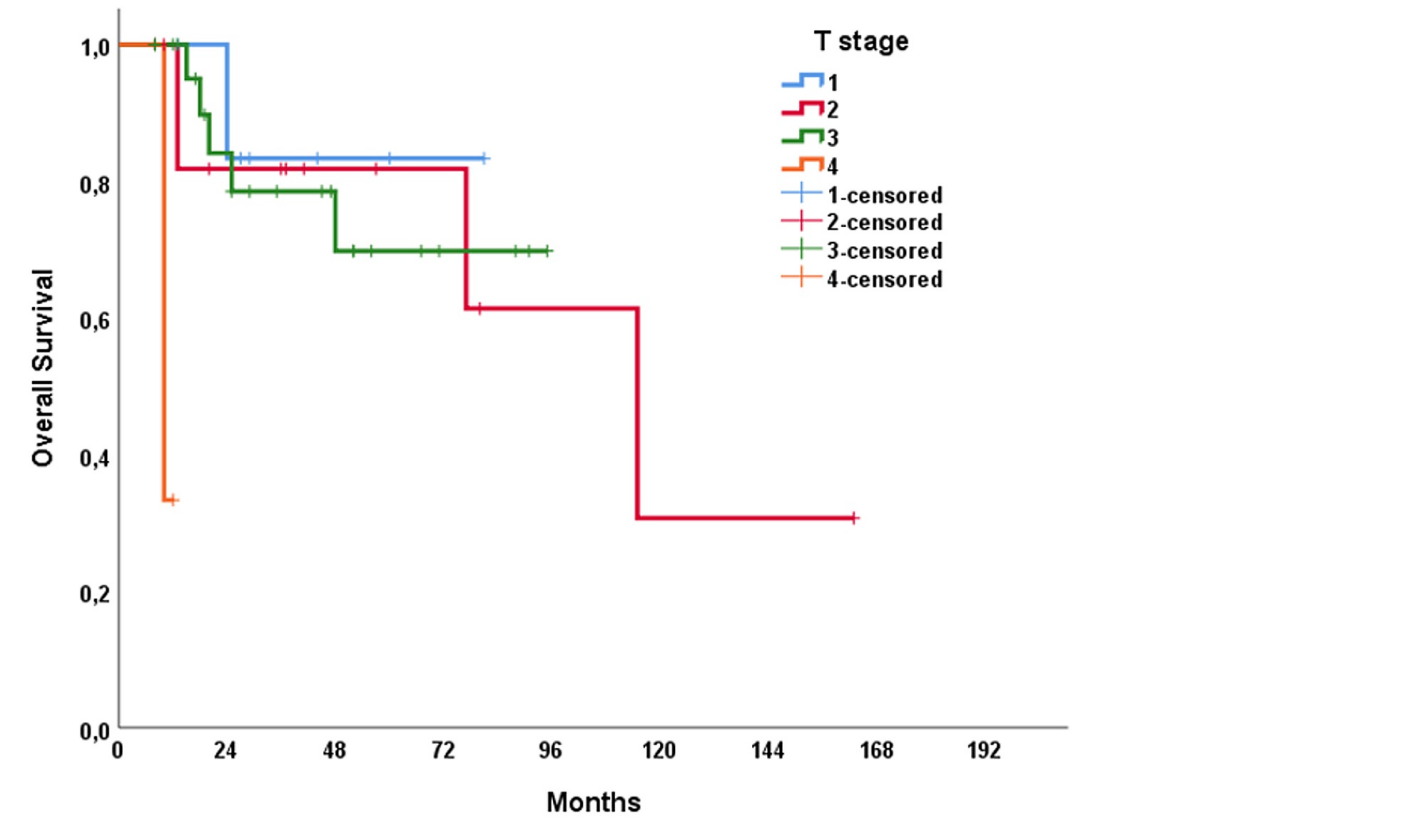
Overall survival by T stage

**Figure 5:**
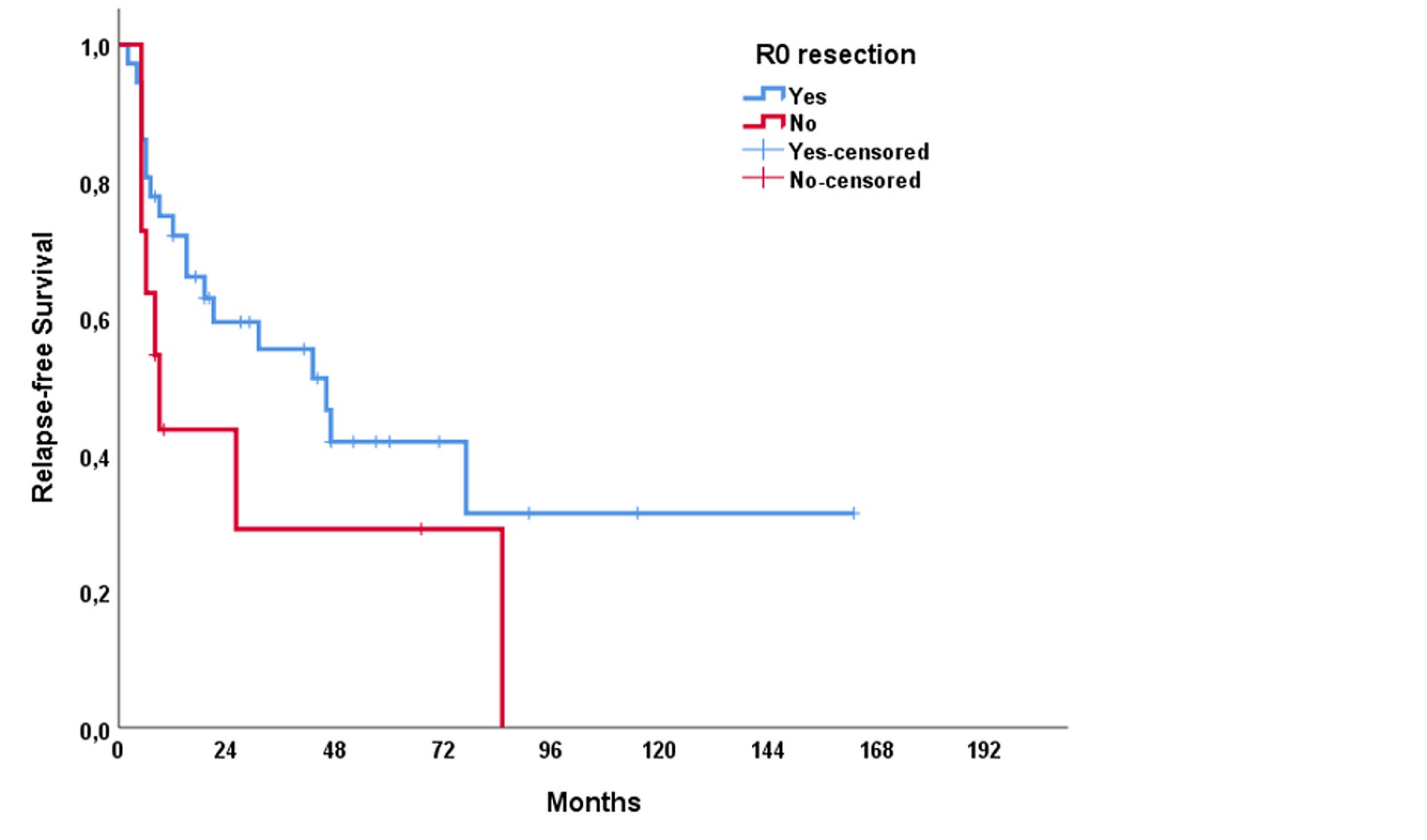
Relapse-free survival by R0 resection

**Figure 6:**
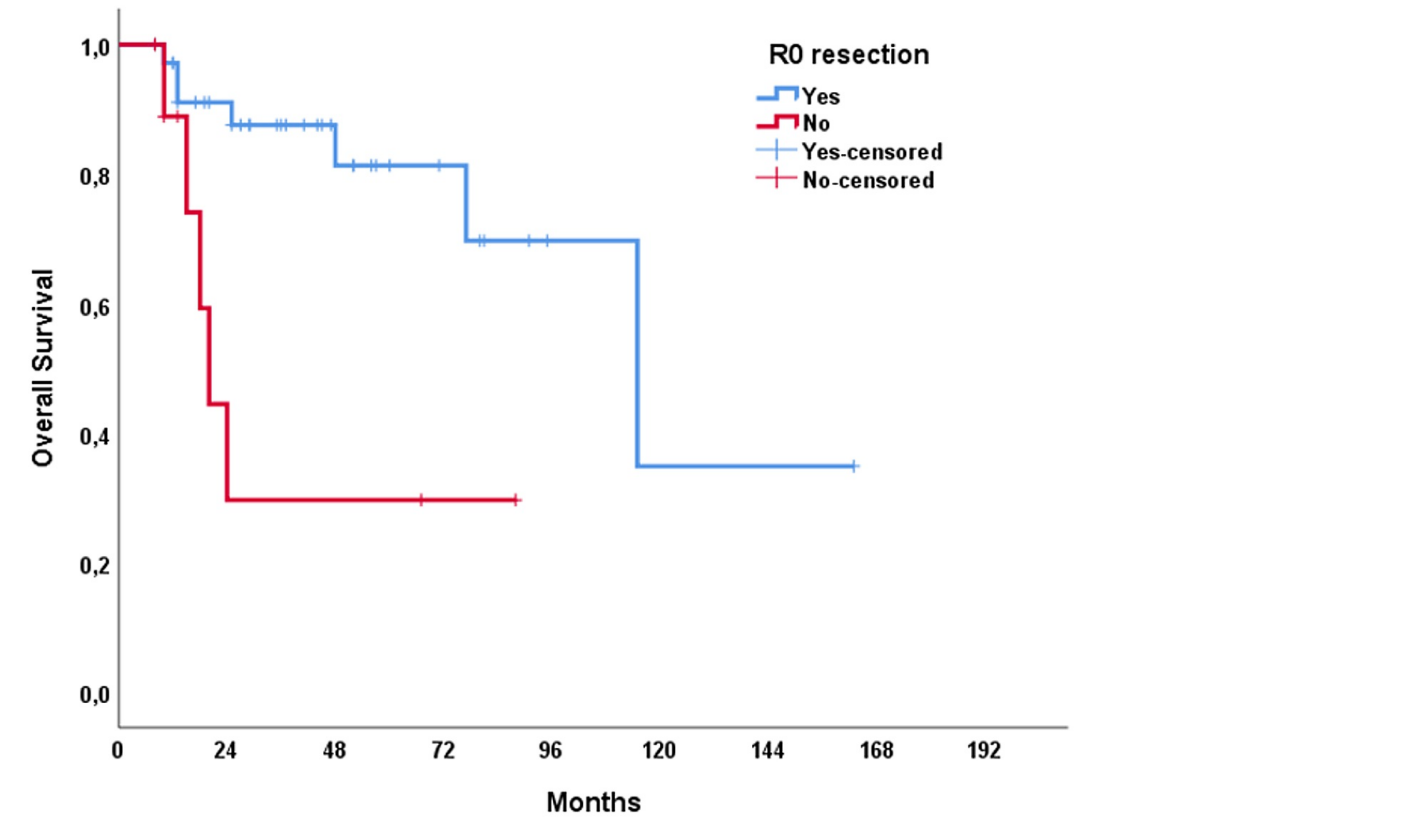
Overall survival by R0 resection

The median RFS of the study population was 31 months (range: 1.0-61.1 months), and the median OS was 115 months (range: 60.8-169.2 months). In univariable analysis, treatment modality showed a significant impact on RFS (p = 0.021); however, no significant association was present with OS (p = 0.137) (Figures 1, 2). The T stage of the tumor did not affect RFS (p = 0.320) but affected OS (p < 0.001) (Figures 3, 4). R0 resection did not showed impact on RFS (p = 0.130) but showed a significant association with OS (p = 0.004) (Figures 5, 6). Tumor grade, gender, necrosis, and lymphovascular invasion did not affect OS and RFS (Table 2).

**Table 2:** Univariable analysis of factors affecting OS and RFS CT, chemotherapy; LVI, lymphovascular ınvasion; NA, not applicable; NR, not reached; OS, overall survival; RFS, relapse-free survival; RT, radiotherapy

	RFS, months (95% CI)	p-Value	OS, months (95% CI)	p-Value
All patients	31 (1.0-61.1)	-	115 (60.8-169.2)	-
Gender				
Male	43 (7.9-78.0)	0.579	77 (24.1-129.9)	0.235
Female	31 (1.0-67.2)		NR	
Tumor grade				
1-2	43 (17.5-68.5)	0.683	NR	0.089
3	15 (1.0-56.5)		77 (1.0-153.0)	
Necrosis				
Yes	31 (0-76.1)	0.928	115 (NA)	0.658
No	26 (0-55.9)		NR	
LVI				
Yes	31 (2.9-59.1)	0.356	115 (19.0-210.9)	0.417
No	77 (0-217.8)		NR	
Tumor stage				
T1	43 (NA)	0.320	NR	<0.001
T2	47 (1.0-107.7)		115 (57.1-172.9)	
T3	15 (1.0-42.1)		NR	
T4	6 (4.4-7.6)		10 (NA)	
R0 resection				
Yes	46 (24.3-67.7)	0.130	115 (60.6-169.4)	0.004
No	9 (4.4-13.5)		20 (15.0-24.9)	
Treatment modality				
Surgery	21 (0-49.9)	0.021	NR	0.137
Surgery + RT	12 (2.2-21.8)		25 (3.5-46.4)	
Surgery + CT	9 (0-26.0)		77 (0-155.2)	
Surgery + RT + CT	85 (NA)		115 (NA)	
Tumor location				
Neck	26 (0-62.3)	0.924	115 (19.3-210.7)	0.997
Scalp/face	19 (0-43.4)		NR	
Supraclavicular	47 (0-110.7)		77 (NA)	
Paranasal sinus	31 (0-68.8)		NR	

Multivariable analysis revealed that T stage of the tumor (hazard ratio [HR]: 3.834; 95% CI: 1.631-9.008; p = 0.002), and treatment with surgery and sequential RT and CT (HR: 0.115; 95% CI: 0.035-0.371; p < 0.001) were independent factors associated with RFS. Only R0 resection (HR: 4.902; 95% CI: 1.301-18.465; p = 0.019) was independently associated OS (Table 3).

**Table 3:** Multivariable analysis of factors affecting OS and RFS CT, chemotherapy; RT, radiotherapy; RFS, relapse-free survival; OS, overall survival

	RFS		OS	
	HR (95% CI)	p-Value	HR (95% CI)	p-Value
T stage				
T1-2	Reference	0.002	Reference	0.204
T3-4	3.834 (1.631-9.008)		2.392 (0.623-9.181)	
Tumor grade				
1-2	-	-	Reference	0.086
3	-		2.945 (0.859-10.099)	
R0 resection				
Yes	Reference	0.203	Reference	0.019
No	1.795 (0.730-4.416)		4.902 (1.301-18.465)	
Treatment modality				
Surgery	Reference		-	-
Surgery + RT	0.675 (0.204-2.235)	0.520	-	
Surgery + CT	0.801 (0.307-2.088)	0.650	-	-
Surgery + RT + CT	0.115 (0.035-0.371)	<0.001	-	-

## Discussion

Soft tissue sarcomas account for about 1% of all malignancies in the head and neck region and 4-10% of all adult sarcomas [12,13]. The primary goal of surgical tumor resection should be obtaining local control with negative margins at the first attempt, preventing increased morbidity, and having the best chance for a possible cure. Around 30% of HNSTSs occur in children. The median age at diagnosis is between 50 and 54 years in all patients, and when pediatric patients are not included, the median age at diagnosis is between 55 and 59 years [14]. The median age in the study population was 51 years (range: 18-85 years). There was a male predominance (66%) in our cohort, similar to the previous series [15]. In our study, the most common histological subtype was synovial sarcoma, whereas we excluded osteogenic sarcomas, non-chemosensitive and non-radiosensitive sarcomas consisting of alveolar soft part sarcomas, dermatofibrosarcoma protuberans, and gastrointestinal stromal tumors in this study. We found that the T3-4 tumor stage was an independent predictor of poor RFS in the multivariable analysis but did not affect OS. In an Italian retrospective cohort of 101 patients, it was demonstrated that AJCC 8 T classification cut-off points were not significantly different on multivariable analysis [16]. Another recent study revealed that the currently used AJCC 8 T classification cut-off points were not prognostic [17]. Positive surgical margins are associated with poor prognosis [18]. In a series of 146 patients with a variety of skull base sarcomas (both of soft tissue and bone), five-year disease-specific survival rates were 77%, 43%, and 36% for those with negative, close (often defined as <1 mm), and positive surgical margins, respectively, and the presence of positive or close margins was the only independent predictor of poor survival in multivariable analysis [19]. Our study showed that R0 resection was a positive predictor factor for OS but did not relate to RFS. In one study of 122 sarcomas of the head or neck, patients with high-grade lesions had significantly worse survival compared with those with low-grade lesions (HR for death: 5.52; 95% CI: 1.51-20.21) [20]. In our study, no correlation was found between tumor grade and OS and RFS. Also, we found that necrosis, lymphovascular invasion, and gender had no effect on OS and RFS. The low number of cases could explain these results.

Recommendations for adjuvant CT and/or RT are made for each case based on multidisciplinary evaluation of all clinical and histopathological features, tumor CT and radiation sensitivity, margin status, and high-risk conditions. Park et al. demonstrated that RT and CT were not associated with improved disease-specific survival or OS [20]. Mattavelli et al. showed that adjuvant RT did not demonstrate a survival benefit [21]. In a recent study, Mahmoud et al. demonstrated that adjuvant RT was associated with improved survival in high-grade HNSTS [22]. Most of our study population received adjuvant RT and CT sequentially. In the multivariable analysis, we showed that adjuvant RT and CT improved RFS. In univariable analysis, treatment modality did not affect OS. Adjuvant only CT or only RT did not demonstrate any survival benefit.

Our study had some limitations. First of all, it is a retrospective analysis of patients from various medical oncology departments. Histopathological evaluations of the patients may vary depending on the experience of institutions. Unfortunately, we could not have had a pathological reevaluation of the paraffin blocks by the same pathologist. Therefore, the lack of a central pathological assessment is another limitation of this study. Our study consisted of a small sample size. We could not have had any molecular evaluation in our patients. On the other hand, our study offers real-life data.

## Conclusions

Our study revealed that R0 resection improved OS, and T3-4 stage of tumor was a negative independent factor for RFS in surgically resected HNSTS patients. The use of sequential CT and RT after resection was associated with a better RFS, which emphasized the importance of multidisciplinary evaluation of the treatment of HNSTS. Randomized prospective studies are needed to determine the adjuvant treatment strategies and prognostic factors of HNSTS. Identification of possible biomarkers may enable us to tailor indications and choice of adjuvant treatment modality in patients with HNSTS.
